# Self-Reported Adverse Drug Reactions, Medication Adherence, and Clinical Outcomes among Major Depressive Disorder Patients in Ethiopia: A Prospective Hospital Based Study

**DOI:** 10.1155/2017/5812817

**Published:** 2017-11-14

**Authors:** Tadesse Melaku Abegaz, Lamessa Melese Sori, Hussien Nurahmed Toleha

**Affiliations:** ^1^Department of Clinical Pharmacy, School of Pharmacy, College of Medicine and Health Sciences, University of Gondar, Gondar, Ethiopia; ^2^Department of Psychiatry, Gondar University Hospital, Gondar, Ethiopia; ^3^Pharmaceutics Unit, Department of Pharmacy, College of Medicine and Health Sciences, Wollo University, Dessie, Ethiopia

## Abstract

**Background:**

There is paucity of data on prevalence of Adverse Drug Reactions (ADRs) and adherence and clinical outcomes of antidepressants. The present study determined the magnitude of ADRs of antidepressants and their impact on the level of adherence and clinical outcome.

**Methods:**

A prospective cross-sectional study was conducted among depression patients from September 2016 to January 2017 at Gondar University Hospital psychiatry clinic. The Naranjo ADR probability scale was employed to assess the ADRs. The rate of medication adherence was determined using Morisky Medication Adherence Measurement Scale-Eight.

**Results:**

Two hundred seventeen patients participated in the study, more than half of them being males (122; 56.2%). More than one-half of the subjects had low adherence to their medications (124; 57.1%) and about 186 (85.7%) of the patients encountered ADR. The most common ADR was weight gain (29; 13.2%). More than one-half (125; 57.6%) of the respondents showed improved clinical outcome. Optimal level of medication adherence decreased the likelihood of poor clinical outcome by 56.8%.

**Conclusion:**

ADRs were more prevalent. However, adherence to medications was very poor in the setup. Long duration of depression negatively affects the rate of adherence. In addition, adherence was found to influence the clinical outcome of depression patients.

## 1. Introduction

Major Depression (MD) is the most common mental health problem in the world. Life time prevalence of 16.2% million among adults has been reported in United States [1]. The scale of global impact of mental illness is substantial, constituting an estimated 7.4% of the world's measurable burden of disease [2]. Major Depressive Disorder (MDD) is the second leading cause of years lived with disability (YLDs) globally and ranks among the four largest contributors to YLDs [2]. In economic terms, a study commissioned by the World Economic Forum concluded that the world encountered a cumulative output loss of $47 trillion between 2011 and 2030 due to noncommunicable diseases and mental illness [3].

In Ethiopia different studies among varying groups of participants reported a range of findings. In the northern part of the country, the burden of depression was found to be about 17.5% among community dwellers [4]. Another study among women attending antenatal care in a teaching referral hospital reported a 23% prevalence [5]. Prevalence of the disease among epileptic patients in northwest Ethiopia was estimated to be 45.2% [6]. A systematic review, which determined a pooled prevalence of the diseases in Ethiopia, reported a prevalence of 6.8% [7]. As to the mortality caused by depression, a population based study determined that the mortality rate of the disease was 3.5% [8]. Etiologies such as psychological, biological, and environmental factors contributed to its prevalence [9].

Once it happened, depression requires a due attention to treat or hold its progression. The management of Major Depressive Disorder (MDD) requires the combination of pharmacologic and nonpharmacologic strategies. Tricyclic antidepressants (TCAs), selective serotonin reuptake inhibitors (SSRIs), and serotonin norepinephrine reuptake inhibitor (SNRIs) medications are the common pharmacologic agents [10]. The coincidence of depression along with other clinical characteristics such as psychotic and bipolar features requires initiation of combination medication which predisposes patients to Adverse Drug Reactions (ADRs). After initiation of antidepressants, a significant number of patients report ADRs. Self-reported ADRs encompass spontaneous reporting or easy probing by health care providers [11]. Different types of ADRs have been reported due to antidepressant. A study among elderly inpatients in Italy demonstrated that cardiovascular and arrhythmic complications and gastrointestinal ADRs were the most common ones. It showed that ADRs were associated with frequency of depression and women were found to suffer higher incidence of depression and mostly affected by ADRs [12].

Though patients report untoward effect of medications, the temporal-relationship of ADR with the drug is established by the utilization of standard tools [13]. ADRs affect the compliance of patients to their medications. A study in the USA reported participants with worrying side-effects tended to be nonadherent to their antidepressant medications [14]. The efficacy of antidepressants, on the other hand, is affected by the rate of adherence to medications. A review article revealed that nonadherence remains a major challenge in achieving good clinical outcomes [15]. Another review reported uncontrolled depression also might lead to medication discontinuation [16]. In addition, factors such sex, age, and duration of illness and side-effects were associated with level of adherence [17]. Hence, medication safety, efficacy, and level of adherence are considered to be interrelated [18].

Despite the interdependency of the above parameters, there is paucity of data that reveal the impact of ADRs on the level of medication adherence in patients with depression in developing setting. Investigation of the magnitude of ADRs, medication adherence, and clinical outcome will provide a clue to design coping strategies against the most deleterious ADRs based on severity and probability of the ADR. In addition, determination of the overall adherence and clinical status of patients would enable evaluating the effectiveness of our interventions. Hence, the present study sought to determine the level of adherence to and clinical outcome of antidepressants and magnitude of their ADRs. In addition, the study has also aimed to identify factors affecting adherence and clinical outcome in depression patients attending psychiatry clinic of referral and teaching hospital.

## 2. Patients and Methods Study Setting and Design

A prospective cross-sectional study was conducted at Gondar University Hospital (GUH) from September 2016 to January 2017. GUH is a teaching referral hospital that serves more than 5 million people in northwest Ethiopia. It has both inpatient and outpatient departments. The medical outpatient department comprised chronic disease clinics including psychiatry unit. The psychiatry clinic provides inpatient service to admitted patients and the outpatients for follow-up of bipolar, depression, substance abuse and schizophrenic patients.

### 2.1. Study Subjects and Population

All depression patients that do have regular visit at GUH constituted our source population. Patients who have been taking antidepressant for at least one month were included in the study.

### 2.2. Study Variables

Level of adherence to antidepressants, patient clinical outcome, and ADRs were our primary end points whereas sociodemographic characteristics of the patient including age, gender, residence, distance from hospital, and disease duration were the independent variables.

### 2.3. Data Collection Procedures

#### 2.3.1. Data Collection Tools

The Naranjo ADR probability scale, which contains ten items, was employed to assess the ADRs due to antidepressants. Based on the scale, ADR is considered to be definite if the score is ≥9, probable if the score is 5–8, possible if the score is 1–4, and doubtful if the score is 0 [19]. The Antidepressant Side-Effect Checklist (ASEC) which was developed by Royal College of Psychiatrists was applied to classify the MSE into mild, moderate, and sever [20]. In addition, the type of ADR reported by the patient was reported from ASEC as all ADRs have been listed in ASEC. Clinical outcomes of patients were assessed by using patient health questionnaire-9 (PHQ-9) developed by Kroenke and Spitzer and adopted to our setup [21]. According to these criteria, patients' status was classified as mild, moderate, moderately sever, and severe depression by presenting nine questions that have bothered patients in the last two weeks. To make the clinical outcome a dichotomous categorical variable, patients were classified as “improved” by combining mild and moderate and “not improved” (moderately severe and severe depression), based on the PHQ-9. The rate of medication adherence (MA) was determined using Morisky Medication Adherence Measurement Scale-Eight (MMAS-8). According to this scale a score less than six indicates low adherence, a score of 6-7 moderate level of adherence, and a score of 8 high level of adherence. In this study, high adherence was considered optimal adherence and low and medium adherence were defined as suboptimal adherence [22]. All tools were translated in Amharic version and showed reliability of more than 80% (Cronbach alpha).

#### 2.3.2. Data Collection Process

Data was collected by clinical psychiatrist using a pretested structured data collection tool. The data collector was trained intensively on contents of questionnaire, data collection methods, and ethical concerns. The data collector utilized a face-to-face interview with patients. Data collection has been conducted in both the inpatient and outpatient clinic of GUH. Patients were requested to report only the one most annoying ADR during the last one month.

### 2.4. Data Analysis

All the statistical data were carried out using Statistical Package for Social Sciences (SPSS), version 21 (SPSS Inc., Cary, NC, USA). Descriptive statistics were presented using means with standard deviation (±SDs) and percentages (%). *P* values < 0.05 were considered significant. Multivariable logistic regression was carried out to determine factors for MA and clinical outcome.

## 3. Results

### 3.1. Sociodemographic Characteristics

**Figure 1:**
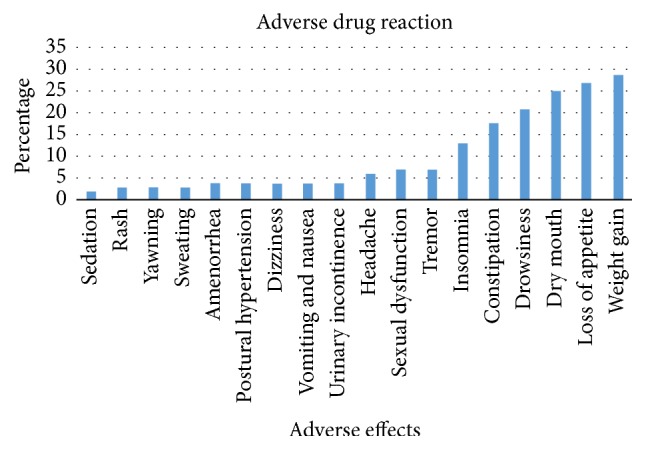
The frequency of patient reported side-effects (*n* = 217).

Two hundred seventy patients participated in the study giving a 100% response rate. More than half of the respondents were males (122; 56.2%). The mean age of the participants was 30.94 ± 8.85. About 125 (57.6%) of them came from urban area. The majority of the patients were farmers and laborers (75; 34.6). Nearly forty percent of them complete some college or university education (91; 41.9%). The mean duration of the disorder was 1.67 ± 0.948 years. The prevalence of comorbid disorder was 13 (6.0%). Diabetes and human immune virus (HIV) were the most common ones. The mean distance in hours from the hospital was 1.027 ± 0.78 hr. Around 125 (57.6%) had improved outcome. The most common prescribed monotherapy medication was amitriptyline (61; 28.1%) followed by fluoxetine (59; 27.2%). Combination of chlorpromazine plus amitriptyline (25; 11.5%) was frequently prescribed dual therapy. More than one-half of 125 (57.6%) patients had concomitant psychiatric illness. The most common comorbid psychiatric condition was MDD with psychotic feature (16.61%) whereas the list coincident was MDD with manic episodes (3.7%) (Table 1, Figure 1).

### 3.2. Level of Medication Adherence

The mean level of adherence was 4.74 ± 2.19 out of eight scores. More than one-half of the subjects had low adherence to their medications (124; 57.1%). Nearly one-third of them had moderate adherence (70; 32.3%). Only 23 (10.6%) of them achieve high adherence level.

### 3.3. Adverse Drug Reaction

**Figure 2:**
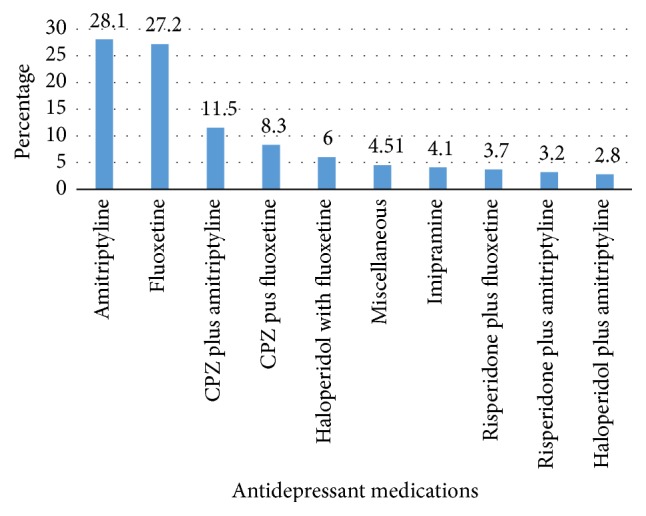
Antidepressant medications.

About 186 (85.7%) of patients encountered ADR. The most common ADR was weight gain (29; 15.59%) followed by loss of appetite (27; 14.52%). Sedation was rarely reported ADR (2; 1.1%) (Figure 2).

### 3.4. ADR Severity Score and Naranjo Probability Scale

According to ASEC majority of the ADRs were moderate (150; 69.1%), followed by severe (33; 15.2) and mild (3; 1.4%). Based on Naranjo score, about 198 (92.2%) ADRs were probable and 19 (8.8%) were possible (Table 2).

### 3.5. Factors Associated with Medication Adherence

According to bivariate analysis, age, education, work, and comorbidity were not found to be correlated with the dependent variable. Patients with long duration of disease (above two years) were 2.5 times more likely to be nonadherence, adjusted odds ratio (AOR): 2.424 [1.185–4.961], and those who came from long distance were found to be five times nonadherent to their medications as compared to short distance, AOR: 5.061 [1.792–14.928]. Patients with concomitant psychiatric illness tended to be more nonadherent, AOR: 2.228 [1.009–4.518] (Table 3).

More than one-half (125; 57.6%) of respondents showed improved outcome. Optimal level of medication adherence decreased the likelihood of poor clinical outcome by 56.8%, AOR: 0.432 [0.201–0.909], whereas female gender increases the likelihood of poor outcome by threefold as compared to males, AOR: 2.919 [1.527–5.279] (Table 4).

## 4. Discussion

Depression is one of the common psychiatric illnesses which requires adequate medical treatment. Initiation of antidepressant along with psychotherapy is expected to alleviate symptoms. However, the clinical outcome depends on patients' persistence with medications with no significant apparent ADRs. The present study assessed three interrelated issues including self-reported ADRs, medication adherence, and clinical outcomes of patients receiving antidepressants. This study has found that ADRs were more prevalent in MDD patients as above eighty percent of the respondents reported ADRs. Nearly twenty types of ADRs were identified. The most common ADR was weight gain which might be due to the prescription of tricyclic antidepressants such as amitriptyline [23]. Moreover, our study has found that the most common prescribed monotherapy medication was amitriptyline (61; 28.1%) followed by fluoxetine (59; 27.2%). Older generation antidepressants are associated with wide range of side-effects and they are commonly prescribed in Ethiopia due to reduced cost. These traditional medications were also given in combination. For instance, combination of chlorpromazine plus amitriptyline (25; 11.5%) was frequently prescribed dual therapy. In addition to different classes of antidepressants, the lack of improvement of the disease might contribute to the weight bearing effect of the disease [24]. The self-reported ADRs including weight gain should be given attention because more than ninety percent of them were probably due to the medications according to the Naranjo ADR probability scale. The underreporting of side-effects such as sexual dysfunction by depression patients could lead to nonadherence and poor improvement which increases suicidal ideation [25]. Even though the majority of them were mild to moderate, it requires reassurance of the patients so as to maintain high medication adherence which could be achieved with collaborative care [26].

In this study, only one-tenth of subjects achieved adequate medication adherence. The rate of adherence was also low among depression patients in Thailand [27]. A ten-year cumulative evidence reported that approximately fifty percent of depression patients discontinue their medications [28]. Sriharsha (2015) has reported that various factors such as duration of therapy and disease were found to influence adherence [29]. Our study has also found that long-standing depression and comorbid psychiatric features increased the chance of nonadherence. Another study from India also identified that nonadherence was higher among female participants which revealed the same finding with the present study [30]. According to a result of patient survey, individual side-effects including weight gain and being unable to orgasm were associated with nonadherence [20, 31]. However, the current study was not able to assess correlation with respect to each type of ADRs due to violation of assumptions of logistic regressions; rather the level of adherence was not found to be associated with the probability and severity of ADRs. Other factors play a pivotal role in predicting adherence in the set. Whenever depression occurs with other unspecified psychiatric features, there might be misdiagnosis of one or more components which could lead to undertreatment and nonadherence [32]. In addition, long duration of disease remains to be an important determinant for poor adherence; this is due to the lack of improvement of the disease which might make patient lose belief in the effectiveness of their medications. Overall, to address these challenges and to achieve optimal medication adherence, application of adherence-enhancement interventions is vital [33]. In addition, adherence to medications could be promoted by the establishment of close communication between the patient and the physician [27].

More than fifty percent of patients attained optimal control of the disease in the present study. In contrast to this, another study reported low rate of remission among major depression patients attending primary care. This might be due to the difference in the level of care, therapeutic alternatives, and scale of measurements of the clinical outcome [34]. In our study, the optimal antidepressant medication adherence decreases the incidence of poor treatment outcome. Gender difference also has been observed in terms of achieving improved outcome. Accordingly, females tended to have poor outcome compared to males. This might be due to undiagnosed past history of sexual assaults among females. According to previous reports sexual assault was considered to be correlated with poor outcome [35]. In addition, biological factors such as postnatal depression might pose significant barriers to treatment outcome among women [36]. In our study clinical outcome was measured only at a single visit; hence the frequency of relapse was not determined. Evidences have indicated that a significant number of patients who achieved remission developed a relapse [37]. Investigation of clinical outcome of more than one visit would enable designing more effective interventions for patients who do not show robust improvement in treatment. These interventions include the implementation of nonpharmacologic psychotherapy that has been shown to provide more benefits over pharmacotherapy alone [38].

Generally, this study highlighted the extent of adherence, clinical outcomes, and prevalence of patient reported ADRs. However, it is limited to single center and the current sample size decreases the power of the study to determine associating factors of adherence and clinical outcomes. The authors call for a large multicenter study which evaluates clinical outcome based on comparative effectiveness of antidepressants and by measuring clinical outcome in subsequent visits.

## 5. Conclusion

The current study identified that patient reported ADRs were highly prevalent among MDD patients, weight gain being the most common. Adherence to medications was very poor in the hospital which was attributed to factors such as long-standing depression, distance from the follow-up clinic, and comorbid psychiatric illness. In addition, clinical outcome of patients was affected by nonadherence to antidepressant medications. The authors recommend that clinicians implement adherence enhancing interventions for their patients. Moreover, further research would establish that ADRs, adherence, and clinical outcome influence each other. Furthermore patients should be asked for ADRs so as to insure the safety of antidepressants.

## Figures and Tables

**Table 1:** Sociodemographic and clinical characteristics of the respondents.

Variables	Frequency *N* (%)
*Age (mean ± SD) *	30.94 ± 8.853
*Male*	122 (56.2%)
*Duration of the disease *	1.67 ± 0.948 yrs
*Urban *	125 (57.6%)
*Education, college and university *	91 (41.9%)
*Occupation, labor *	75 (34.6)
*Distance (mean ± SD)*	1.027 ± 0.78 hr
*Comorbidity *	13 (6.0%)
*Comorbid psychiatric features (n = 125)*	
Depression with psychotic feature	40 (18.4%)
Depression with GAD	36 (16.61%)
Depression with otherwise not specified characteristics	17 (7.8%)
Depression with manic episode	32 (14.7%)

CPZ: chlorpromazine, generalized anxiety disorder.

**Table 2:** Severity and probability of ADRs.

Variables	*N* (%)
Severity	
Mild	3 (1.4)
Moderate	150 (69.1)
Sever	33 (15.2)
Probability	
Probable	198 (92.2)
Possible	19 (8.8)

**Table 3:** Factors associated with medication adherence.

Variable	Level of adherence		Crude OR [95% CI]	Adjusted OR [95% CI]
	Optimal	Suboptimal		
Sex				
Male	55 (25.34)	67 (30.88)	1	1
Female	38 (17.51)	57 (26.26)	1.231 [0.715–2.121]	1.103 [0.551–2.208]
Residence				
Urban	72 (33.18)	53 (24.42)	1	1
Rural	21 (9.68)	71 (42.72)	4.593 [2.515–8.389]^*∗*^	2.008 [0.933–4.32]
Disease duration				
Less than two years	51 (23.03)	41 (18.89)	1	1
More than two years	42 (19.35)	83 (9.68)	2.458 [4.413–4.227]^*∗∗*^	2.424 [1.185–4.961]^*∗*^
Distance				
<2 hours	82 (37.78)	71 (32.72)	1	1
≥2 hours	11 (5.10)	53 (24.42)	5.565 [2.701–11.466]^*∗∗*^	5.061 [1.792–14.928]^*∗*^
Other psychiatric illness				
Yes	40 (18.43)	85 (39.17)	1	1
No	53 (24.42)	39 (17.97)	2.888 [1.652–5.049]^*∗∗*^	2.228 [1.009–4.518]^*∗*^
Naranjo score				
Possible	12 (5.53%)	7 (3.22%)	1	1
Probable	56 (28.80%)	112 (51.61%)	3.429 [1.279–9.188]^*∗*^	2.838 [0.923–8.701]
Severity of ADR				
Moderate	59 (27.18%)	91 (41.93)	1	1
Sever	8 (3.69%)	28 (12.90)	2.269 [0.969–5.319]	1.172 [0.422–3.254]

^*∗*^Statistically significant at 0.05. ^*∗∗*^Significant at 0.01.

**Table 4:** Factors associated with the clinical outcome.

Variable	Treatment outcome		Crude OR [95% CI]	Adjusted OR [95% CI]
	Improved (125; 57.6%)	Unimproved (92; 42.4%)		
Sex				
Male	83 (38.25)	39 (17.97)	1	1
Female	42 (19.35)	53 (24.42)	2.686 [1.541–4.681]^*∗∗*^	2.919 [1.527–5.279]^*∗∗*^
Residence				
Urban	74 (34.01)	51 (23.51)	1	1
Rural	51 (23.50)	41 (18.89)	1.166 [0.677–2.010]	0.812 [0.380–1.734]
Disease duration				
Less than two years	41 (18.89)	51 (23.50)	1	1
More than two years	84 (15.47)	41 (18.89)	0.392 [0.225–1.684]	0.249 [0.122–1.508]
Distance				
<2 hours	94 (43.32)	59 (27.19)	1	1
≥2 hours	31 (14.29)	33 (15.21)	1.333 [0.49–2.686]	1.367 [0.603–3.099]
Other psychiatric illnesses				
Yes	68 (43.31)	57 (26.26)	1	1
No	57 (26.26)	35 (16.13)	1.365 [0.789–2.363]	0.824 [0.414–1.642]
Naranjo score				
Possible	10 (4.60)	9 (4.15)	1	Ref
Probable	91 (41.93)	77 (35.48)	0.940 [0.363–2.432]	0.898 [0.303–2.669]
Level of adherence				
Optimal	62 (28.57)	31 (14.28)	0.37 [0.110–5.379]^*∗*^	0.432 [0.201–0.909]^*∗*^
Suboptimal	63 (29.03)	61 (28.11)	1	1
Severity of ADR				
Moderate	86 (39.63)	64 (29.49)	1	1
Sever	14 (6.45)	22 (10.14)	2.112 [1.003–4.444]^*∗*^	2.133 [0.912–4.990]

^*∗*^Statistically significant at 0.05. ^*∗∗*^Significant at 0.01.

## Abbreviations

ADR: Adverse Drug Reaction
AOR: Adjusted odds ratio
ASEC: Antidepressant Side-Effect Check list
COR: Crude Odds Ratio
GUH: Gondar University Hospital
MA: Medication adherence
MDD: Major Depressive Disorder
MMAS-8: Morisky Medication Adherence Scale-Eight
MSE: Medication Side-Effect
PHQ-9: Patient Health Questionnaire-Nine
PR-ADR: Patient Reported Adverse Drug Reaction
SNRI: Serotonin norepinephrine reuptake inhibitors
SSRI: Selective serotonin reuptake inhibitor
TCA: Tricyclic antidepressant
YLD: Years lived with disability.

## Additional Points

*Availability of Data and Material*. Supplementary data could be obtained from the corresponding author upon request.
