# Corrigendum to “Self-Reported Adverse Drug Reactions, Medication Adherence, and Clinical Outcomes among Major Depressive Disorder Patients in Ethiopia: A Prospective Hospital Based Study”

**DOI:** 10.1155/2018/9274278

**Published:** 2018-02-20

**Authors:** Tadesse Melaku Abegaz, Lamesa Melese Sori, Hussien Nurahmed Toleha

**Affiliations:** ^1^Department of Clinical Pharmacy, School of Pharmacy, College of Medicine and Health Sciences, University of Gondar, Gondar, Ethiopia; ^2^Department of Psychiatry, Gondar University Hospital, Gondar, Ethiopia; ^3^Pharmaceutics Unit, Department of Pharmacy, College of Medicine and Health Sciences, Wollo University, Dessie, Ethiopia

In the article titled “Self-Reported Adverse Drug Reactions, Medication Adherence, and Clinical Outcomes among Major Depressive Disorder Patients in Ethiopia: A Prospective Hospital Based Study” [1], the name of the second author was given incorrectly as Lamessa Melese Sori. The author's name should have been written as Lamesa Melese Sori. The revised author list is shown above.
